# Supplementary material for: Artificial intelligence for online characterization of ultrashort X-ray free-electron laser pulses
Source: Sci Rep. 2022 Oct 24;12:17809. doi: 10.1038/s41598-022-21646-x (PMC9592592; doi:10.1038/s41598-022-21646-x)
Supplement: Supplementary file 1 — Supplementary Information. [file 41598_2022_21646_MOESM1_ESM.pdf]

Artificial intelligence for online characterization of ultrashort X-ray free-electron laser pulses

Kristina Dingel[a,f,1,*], Thorsten Otto[a,1], Lutz Marder[b,f], Lars Funke[e], Arne Held[e], Sara Savio[e], Andreas Hans[b,f], Gregor Hartmann[c,f], David Meier[a,c,f], Jens Viefhaus[c,f], Bernhard Sick[a,f], Arno Ehresmann[b,f], Markus Ilchen[g,d,b], and Wolfram Helml[e]

[*]kristina.dingel@uni-kassel.de
[a]Intelligent Embedded Systems, University of Kassel, Wilhelmshöher Allee 73, 34121 Kassel, Germany
[b]Institute for Physics and CINSaT, University of Kassel, Heinrich-Plett-Straße 40, 34132 Kassel, Germany
[c]Helmholtz-Zentrum Berlin für Materialien und Energie, Hahn-Meitner-Platz 1, 14109 Berlin, Germany
[d]European XFEL GmbH, Holzkoppel 4, 22869 Schenefeld, Germany
[e]Technische Universität Dortmund, Fakultät Physik, Maria-Goeppert-Mayer-Straße 2, 44227 Dortmund, Germany.
[f]Artificial Intelligence Methods for Experiment Design (AIM-ED), Joint Lab Helmholtzzentrum für Materialien und Energie, Berlin (HZB) and University of Kassel
[g]Deutsches Elektronen-Synchrotron DESY, Notkestr. 85, 22607 Hamburg, Germany
[1]these authors contributed equally to this work

ABSTRACT

X-ray free-electron lasers (XFELs) as the world's brightest light sources provide ultrashort X-ray pulses with a duration typically in the order of femtoseconds. Recently, they have approached and entered the attosecond regime, which holds new promises for single-molecule imaging and studying nonlinear and ultrafast phenomena such as localized electron dynamics. The technological evolution of XFELs toward well-controllable light sources for precise metrology of ultrafast processes has been, however, hampered by the diagnostic capabilities for characterizing X-ray pulses at the attosecond frontier. In this regard, the spectroscopic technique of photoelectron angular streaking has successfully proven how to non-destructively retrieve the exact time–energy structure of XFEL pulses on a single-shot basis. By using artificial intelligence techniques, in particular convolutional neural networks, we here show how this technique can be leveraged from its proof-of-principle stage toward routine diagnostics even at high-repetition-rate XFELs, thus enhancing and refining their scientific accessibility in all related disciplines.

Supplementary Information

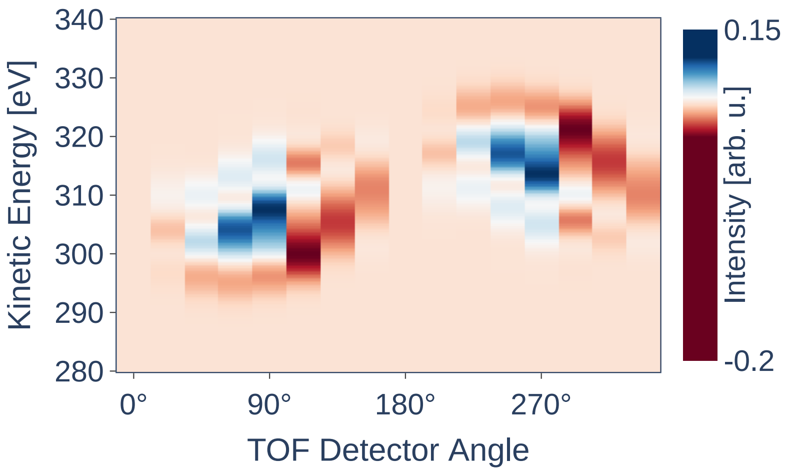

Supplementary Figure 1. Difference between detector images in Fig. 2 (f) and (g). On closer inspection, it is evident that the intensities shift after adding a pulse structure.

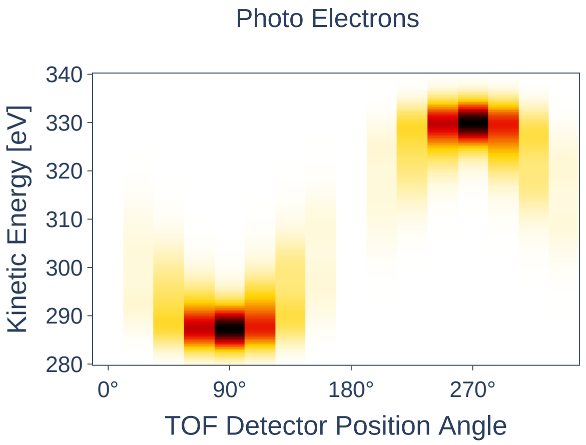

(a) NN estimate (4.8 fs) of the FWHM pulse duration target (4.9 fs) with a large kick (22.5 eV).

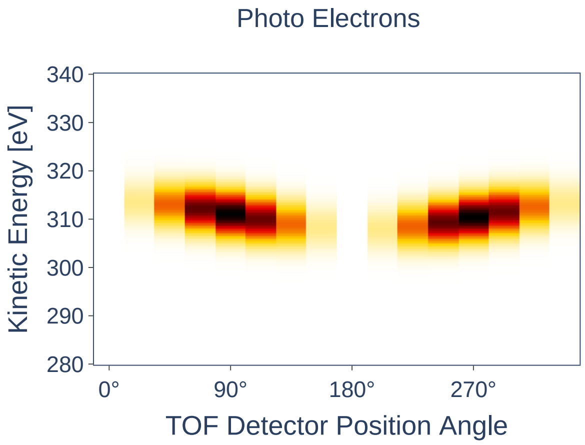

(b) NN estimate (6.3 fs) of the FWHM pulse duration target (4.9 fs) with a small kick (3.0 eV).

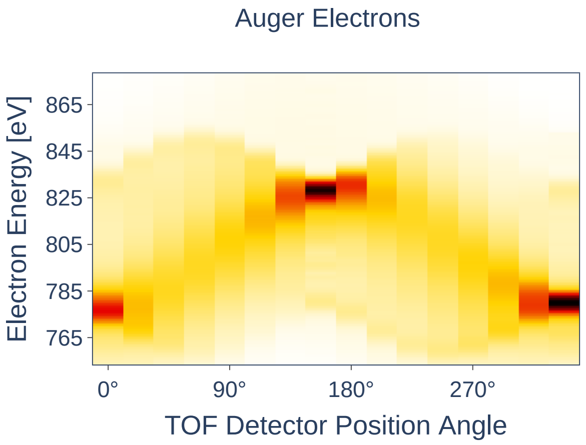

(c) NN estimate (7.0 fs) of the Auger decay time target (7.0 fs) with a large kick (22.5 eV).

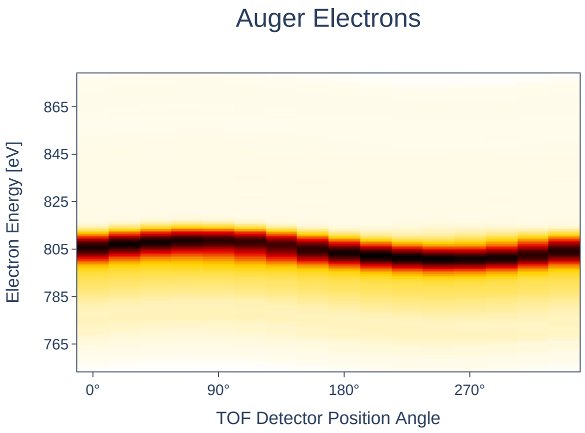

(d) NN estimate (7.6 fs) of the Auger decay time target (7.0 fs) with a small kick (3.0 eV).

Supplementary Figure 2. Exemplary selection of FWHM pulse duration and Auger decay time predictions with large and small kick value, respectively.

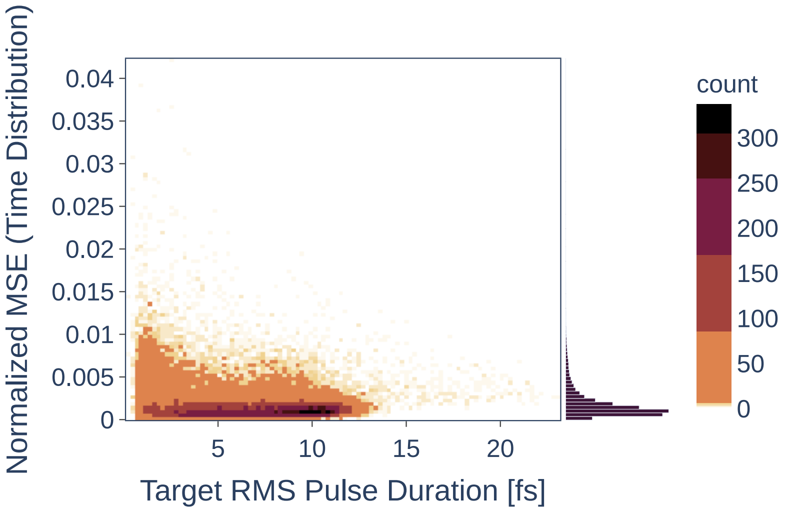

Supplementary Figure 3. Mean squared error of the predicted pulse structure with regard to the true pulse structure, normalized to the respective pulse's duration, shown as a function of the true pulse structure's RMS pulse duration.

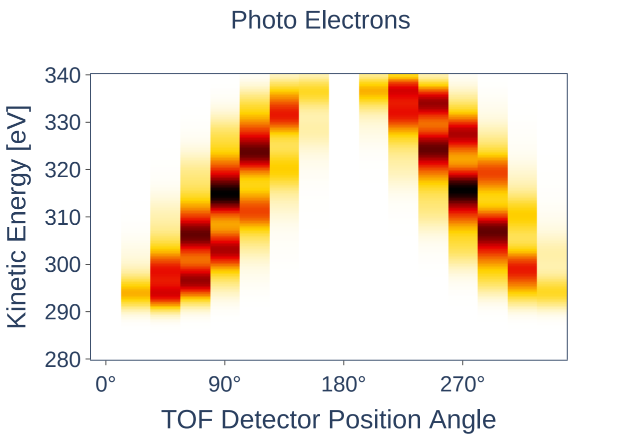

(a) Estimate (22.52 eV) of the target kick label (22.50 eV). The difference is 0.02 eV.

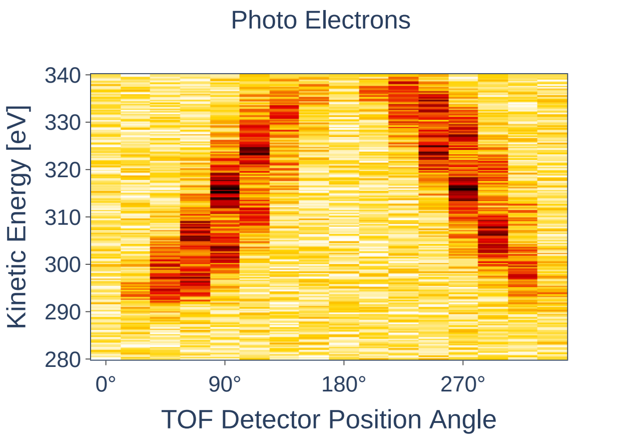

(b) Estimate (22.29 eV) of the target kick label (22.50 eV). The difference is 0.21 eV.

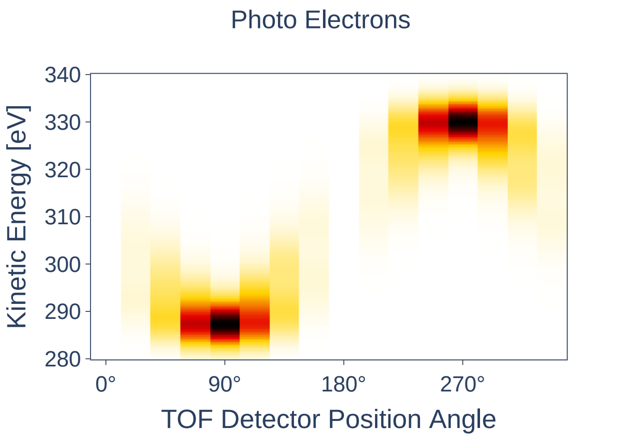

(c) Estimate (4.80 fs) of the target FWHM pulse duration label (4.85 fs). The difference is 0.05 fs.

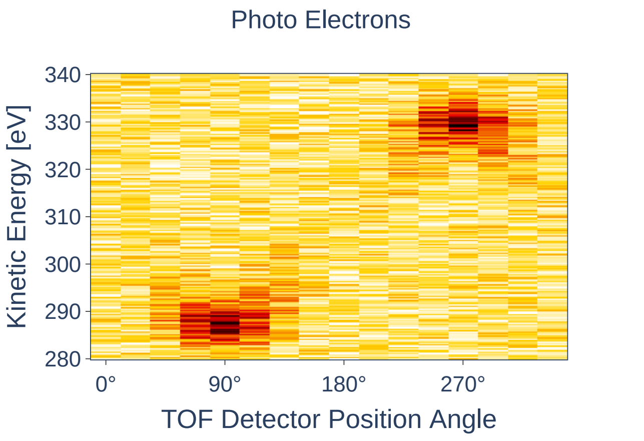

(d) Estimate (5.06 fs) of the target FWHM pulse duration label (4.85 fs). The difference is 0.19 fs.

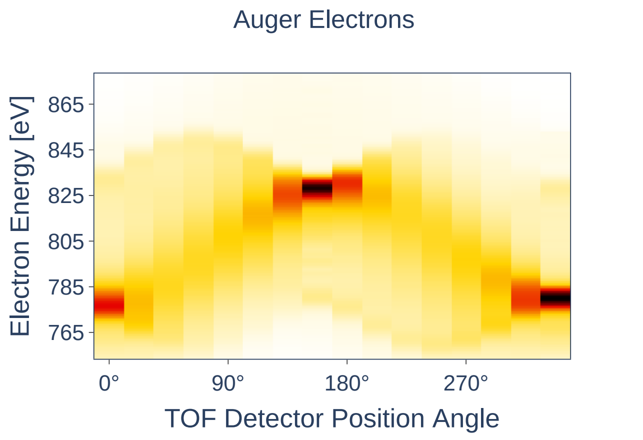

(e) Estimate (7.03 fs) of the target decay label (7.0 fs). The difference is 0.03 fs.

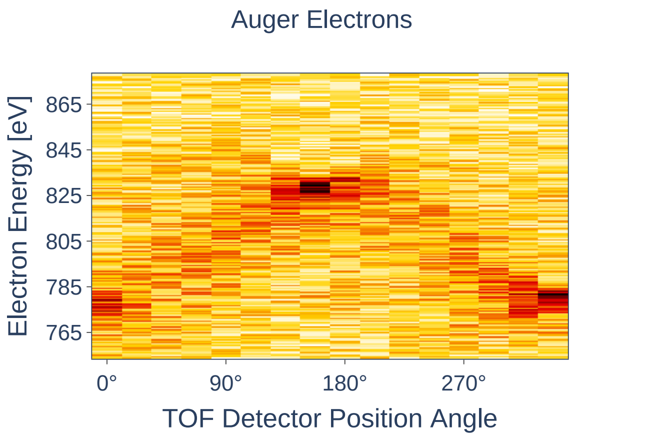

(f) Estimate (7.26 fs) of the target decay label (7.0 fs). The difference is 0.26 fs.

Supplementary Figure 4. The influence of noise on the respective label estimate regarding one sample. (a), (c), (e) display estimates on data without additional noise. (b), (d), (f) show estimates of data with ± 30 % noise added.
